# Isolation and identification of *Vibrio parahaemolyticus* from seawater and sediment samples in the southern coast of the Caspian Sea

**DOI:** 10.1007/s00580-012-1583-6

**Published:** 2012-08-08

**Authors:** Majid Alipour, Khosro Issazadeh, Javad Soleimani

**Affiliations:** 1 Department of Microbiology, Islamic Azad University (IAU), Babol Branch, Babol Iran; 2 Department of Microbiology, Islamic Azad University (IAU), Lahyjan Branch, Lahyjan Iran

**Keywords:** *Vibrio parahaemolyticus*, Seawater, Sediment, Acute gastroenteritis

## Abstract

The objectives of this study were to investigate the occurrence of *Vibrio parahaemolyticus* in the seawater and its sediment by molecular techniques and conventional microbiological methods. Of 300 samples analyzed, 20.3 % was recorded positive for *V. parahaemolyticus*. Of the 62 strains isolated, 26 (8.3 %) were obtained from the seawater samples, and 36 (12 %); from sediments. Only three strains (4.83 %) showed hemolytic activity in Wagatsuma agar. The results of this study demonstrated the presence of *V. parahaemolyticus* in the southern coast of the Caspian Sea (Northern Iran). Furthermore, the PCR approach proved useful for reliable confirmation of species identification. *V. parahaemolyticus* is an important human pathogen responsible for food-borne gastroenteritis worldwide. These findings indicated the potential sanitary risk associated with the presence of pathogenic *V. parahaemolyticus* in the Caspian Sea.

## Introduction

*Vibrio parahaemolyticus* is a human pathogen that is widely distributed in the marine environments. It has a single polar flagellum and is motile when grown in liquid medium (Broberg et al. 2011). *V. parahaemolyticus* is a marine bacterium easily recovered from estuarine and coastal waters worldwide, as well as from seawater (Hayat et al. 2006). *V. parahaemolyticus* requires presence of salinity with optimum levels of 1–3 % to survive and multiply, which is in the range of 0.8–3 % levels commonly found in marine environments (Yeung and Boor 2004). *V. parahaemolyticus* causes three major syndromes of clinical illness, i.e., gastroenteritis, wound infections, and septicemia. Gastrointestinal illness caused by *V. parahaemolyticus* is typically accompanied by symptoms including vomiting, diarrhea, headache, nausea, low-grade fever, and abdominal cramps, and is commonly self-limiting. The mean incubation period for *V. parahaemolyticus* infection is 15 h (Nair et al. 2007). Clinical isolates of *V. parahaemolyticus* most often produce either the thermostable direct hemolysin (TDH) or TDH-related hemolysin (TRH) encoded by *tdh* and *trh* genes, respectively. However, only bacteria producing virulence factors, i.e., TDH and/or TRH, are considered to be pathogenic and can cause acute gastroenteritis (or, more rarely, invasive septicemia) (Bisha et al. 2012). TDH is capable of producing β hemolysis on Wagatsuma agar which is called Kanagawa phenomenon (KP). Most of the strains (90 %) isolated from clinical cases show this type of hemolysis, while only 1–2 % of the strains of environmental origin are KP positive (Drake et al. 2007). TRH is another virulence factor that has been discovered in clinical strains lacking *tdh* gene. TRH is a 23-kDa protein, immunologically related to TDH and environmental strains producing this gene, and produces urease (Pal and Das 2010). Epidemiological studies have revealed an association between the Kanagawa phenomenon and gastroenteritis (Okuda et al. 1997). The objectives of the present study are, first, to determine the prevalence of total and pathogenic *V. parahaemolyticus* in seawater and sediment in the southern coast of the Caspian Sea and, second, to investigate the relationships with water temperature and salinity. The strains isolated were screened for hemolytic activity and for the presence of the *tdh* and *trh* genes. A limitation of the research was the long distance between the sampling sites and the laboratory.

## Materials and methods

**Fig. 1:**
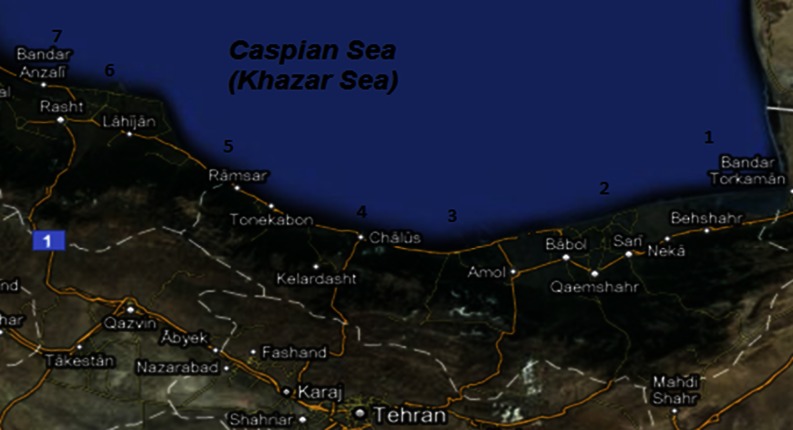
Area of study and locations of the sampling sites in the southern coast of the Caspian Sea (Northern Iran). Site *1* Bandar Torkaman, site *2* Sari, site *3* Noor, site *4* Chalus, site *5* Ramsar, site *6* Chamkhaleh, site *7* Bandar Anzali

The seawater samples and sediments were collected at a depth of 50 and 10–20 cm, respectively, by aseptic techniques. Of the 300 total samples, 150 seawater and 150 sediment samples were collected from seven different sites in the Caspian Sea in April, May, June, July, and August (Fig. 1).

The samples were placed in sealed containers with dry ice and transported frozen to the laboratory for analysis. The time between sample collection and analysis was approximately 24 h, while sampling, water temperature, and salinity were measured.

### *V. parahaemolyticus* isolation and identification

Twenty-five milliliters of seawater samples and 25 g of sediment samples were added to 225 ml of alkaline peptone water (APW) containing 1 % NaCl (pH 8.6), and 10^−2^ and 10^−3^ APW dilutions were prepared in duplicate and incubated at 37 °C for 6–24 h. Enrichment broth was streaked onto thiosulfate-citrate-bile salts-sucrose agar plates and incubated at 37 °C for 18–24 h. The green or blue-green colonies were presumptively selected as *V. parahaemolyticus* colonies and transferred to trypticase soy agar plate containing 3 % NaCl. After incubation at 37 °C for 24 h, the isolates were tested using conventional bacterial methods, including Gram's staining, culture sulfide indole motility and triple sugar iron tests media, cytochrome oxidase activity tests, lysine iron agar tests, urea tests, and tests for arabinose, lactose, mannitol, mannose, and sucrose fermentation (Colakoglu et al. 2006).

### Hemolytic activity

Kanagawa test was performed using Wagatsuma agar supplemented with 5 % human erythrocytes. Each plate was inoculated with *V. parahaemolyticus* strains. Positive reactions were recorded as a zone of β hemolysis surrounding the spot of growth on the human blood plate. The interpretation times for the test were 24, 48, and 72 h (Canizalez-Roman et al. 2011).

### DNA purification

*V. parahaemolyticus* strains were seeded in Luria–Bertani agar supplemented with 1 % NaCl and incubated at 37 °C for 24 h. Five colonies were selected, resuspended in 100 μl of filtered sterile distilled water, and boiled for 15–20 min to liberate the nucleic acid (Cabrera-Garcia et al. 2004).

### Confirmation of strains by polymerase chain reaction

The *tdh* and *trh* genes were amplified with the following primer sets: 5′-GTAAAGGTCTCTGACTTTTGGAC-3′ and 5^′^-TGGAATAGAACCTTCATCTTCACC-3′ for *tdh*, and 5′-TTGGCTTCGATATTTTCAGTATCT-3′ and 5′-CATAACAAACATATGCCCATTTCCC-3′ for *trh* (Tada et al. 1992). These primer sets produced 251- and 373-bp amplicons, respectively. The reaction mixtures (final volume, 25 μl) contained 3 μl of the solution containing DNA, 2.5 μl of 10× reaction buffer, 4 μl of 25 mM MgCl2, 1 μl of *Taq* polymerase (5 U/μl), 4 μl of deoxynucleoside triphosphates (1 Mmol), 1 μl of each primer (20 pmol), and 8.5 μl of distilled water. The reactions were performed as follows: initial denaturation at 94 °C for 1 min, followed by 35 cycles of denaturation at 94 °C for 1 min, annealing at 55 °C for 1 min, extension at 72 °C for 1 min, and a final extension at 72 °C for 7 min. Positive and negative DNA controls were included in all assays. The amplified products were separated by electrophoresis in ethidium bromide-stained 2 % agarose gels in Tris–borate–EDTA buffer at 120 V for 30 min. The gels were visualized with a UV transilluminator.

## Results

A total of 300 samples were analyzed; these samples included 150 seawater and 150 sediment samples. Overall, *V. parahaemolyticus* strains were isolated from 20.3 % of the samples (Table 1). The highest isolation rate of *V. parahaemolyticus* was observed in August, with a total of 23 positive samples (28.3 %). The temperatures ranged between 34 and 39 °C during the sampling period. By contrast, the lowest isolation rate of *V. parahaemolyticus* was observed in April, with a total of 3 positive samples (5 %). The temperature of the seawater ranged from 10 to 15 °C during the sampling period. Salinity was highest in summer (19 ppt), whereas the minimum levels were recorded during the spring (2 ppt). The average salinity was 10.5 ppt during the sampling period.

**Table 1:** Occurrence and the frequency of *V. parahaemolyticus* in the seawater and sediment

Month	April						May						June						July						August						Total frequency	
Source	W		S		W + S		W		S		W + S		W		S		W + S		W		S		W + S		W		S		W + S			
Sites	Frequency																															
	N	%	N	%	N	%	N	%	N	%	N	%	N	%	N	%	N	%	N	%	N	%	N	%	N	%	N	%	N	%	N	%
Site 1	–	–	1	1.6	1	1.6	1	1.6	1	1.6	2	3.3	–	–	–	–	–	–	–	–	2	3.6	2	3.6	1	1.6	3	5	4	6.6	9	3
Site 2	–	–	1	1.6	1	1.6	–	–	–	–	–	–	1	1.6	1	1.6	2	3.3	–	–	1	1.6	1	1.6	–	–	2	3.6	2	3.6	6	2
Site 3	–	–	–	–	–	–	–	–	1	1.6	1	1.6	–	–	2	3.3	2	3.3	1	1.6	1	1.6	2	3.6	–	–	2	3.6	2	3.6	7	2.3
Site 4	1	1.6	–	–	1	1.6	1	1.6	1	1.6	2	3.3	–	–	1	1.6	–	–	3	5	2	3.6	5	8.3	2	3.6	2	3.6	4	6.6	13	4.3
Site 5	–	–	–	–	–	–	–	–	1	1.6	1	1.6	1	1.6	1	1.6	2	3.3	3	5	1	1.6	4	6.6	4	6.6	2	3.6	6	10	13	4.3
Site 6	–	–	–	–	–	–	–	–	–	–	–	–	1	1.6	1	1.6	2	3.3	–	–	–	–	–	–	1	1.6	1	1.6	2	3.6	4	1.3
Site 7	–	–	–	–	–	–	1	1.6	1	1.6	2	3.3	2	3.3	1	1.6	3	5	–	–	2	3.6	2	3.6	2	3.6	1	1.6	3	5	10	3.3
Total	1	1.6	2	3.3	3	5	3	5	5	8.3	8	13.3	5	8.3	7	11.6	11	18.3	7	11.6	9	15	16	26.6	10	16.6	13	21.6	23	28.3	62	20.3

*W* water, *S* sediment

The highest prevalence of *V. parahaemolyticus* was detected at sampling sites 4 (4.3 %) and 5 (4.3 %). Of the 62 strains isolated, 26 (8.3 %) were obtained from seawater samples and 36 (12 %) from sediments. Only three strains (4.83 %) showed hemolytic activity in Wagatsuma agar. Only two of the 62 strains (2.53 %) amplified the 250-bp *tdh* gene fragment, which correlated with positive hemolytic activity. The *trh* gene fragment was amplified in four (5.06 %) of the strains analyzed.

## Discussion

In the present study, we demonstrated the presence of *V. parahaemolyticus* in 20.3 % of the seawater and sediment samples analyzed. This study was the first research to investigate the isolation and distribution of this pathogen in the southern coast of the Caspian Sea. Cabrera-Garcia et al. (2004) reported that 15 % of the seawater samples contained *V. parahaemolyticus*. This pathogen has also been isolated in Canada (Kelly and Stroh 1988), France (Hervio-Heath et al. 2002), Asia (Alam et al. 2002), and the USA (DePaola et al. 2003). In the present study, of the 62 strains isolated, 26 (8.3 %) were obtained from seawater samples and 36 (12 %) from sediments. Velazquez-Roman et al. (2012) have reported that of the 144 strains isolated, 35 % was obtained from seawater samples and 16 % from sediment. Thus, it is speculated that the observed prevalence may also be produced by seasonal variation of the *V. parahaemolyticus* population due to salt and oxygen concentrations, interactions with the plankton, the presence of sediment, and the organic matter in the suspension, fish, and seafood. In one study, it was observed that the maximum probability of *V. parahaemolyticus* detection was around 25 ppt salinity (Martinez-Urtaza et al. 2008). The other reason for the low prevalence of *V. parahaemolyticus* in the southern coast of the Caspian Sea (the average salinity is 10.5 ppt) is its lower salinity than other seas. The distribution of *V. parahaemolyticus* in the marine environments is known to be related to the water temperatures. Studies have shown that the organism was rarely detected in seawater until water temperatures rose to 15 °C or higher. Ecological study of *V. parahaemolyticus* in the Chesapeake Bay of Maryland (USA) found that the organism survived in sediment during the winter and was released from sediment into water column when water temperatures rose to 14 °C in late spring or early summer (Kaneko and Colwell 1978). The isolation rate of *V. parahaemolyticus* was high (28.3 %) in August because a higher rate of evaporation results in high salinity. In one study, it was observed that only two pathogenic *V. parahaemolyticus* were confirmed in environmental samples. The extremely low presence of pathogenic populations of *V. parahaemolyticus* in environmental samples is a constant characteristic in most of the investigations carried out in different regions of the world (Martinez-Urtaza et al. 2008). The results of current investigation are consistent with previous reports. We observed positive *tdh* gene amplification in two of the 62 strains analyzed, which was correlated with the positive hemolytic activity in Wagatsuma agar (KP^+^). Virtually, all clinical isolates of *V. parahaemolyticus* are KP^+^, whereas only 1–2 % of environmental strains are KP^+^ (Drake et al. 2007). This implies that there is a source of human fecal contamination in the seawater and sediment of the southern coast of the Caspian Sea. This information may be important for preventing sanitary problems that may affect the health of the population. In conclusion, this study provided new information on the abundance of *V. parahaemolyticus* in seawater and sediment samples in the southern coast of the Caspian Sea. The data presented in the present study show the presence of pathogenic *V. parahaemolyticus* and suggest the need for a study of such organism in seafood in the Caspian Sea.

## References

[CR1] Alam MJ, Tomochika KI, Miyoshi SI (2002). Environmental investigation of potentially pathogenic *Vibrio parahaemolyticus* in the Seto-Inland Sea, Japan. FEMS Microbiol Lett.

[CR2] Bisha B, Simonson J, Janes M (2012). A review of the current status of cultural and rapid detection of *Vibrio parahaemolyticus*. Int J Food Sci Technol.

[CR3] Broberg CA, Calder TJ, Orth K (2011). *Vibrio parahaemolyticus* cell biology and pathogenicity determinants. Microbes Infect.

[CR4] Cabrera-Garcia ME, Vazquez-Salinas C, Quinones-Ramirez EI (2004). Serologic and molecular characterization of *Vibrio parahaemolyticus* strains isolated from seawater and fish products of the Gulf of Mexico. Appl Environ Microbiol.

[CR5] Canizalez-Roman A, Flores-Villasenor H, Zazueta-Beltran J (2011). Comparative evaluation of a chromogenic agar medium–PCR protocol with a conventional method for isolation of *Vibrio parahaemolyticus* strains from environmental and clinical samples. Can J Microbiol.

[CR6] Colakoglu FA, Sarmasik A, Koseoglu B (2005). Occurrence of *Vibrio* spp. and *Aeromonas* spp. in shellfish harvested off Dardanelles cost of Turkey. Food Control.

[CR7] DePaola A, Nordstrom JL, Bowers JC (2003). Seasonal abundance of total and pathogenic *Vibrio parahaemolyticus* in Alabama oysters. Appl Environ Microbiol.

[CR8] Drake SL, DePaola A, Jaykus LA (2007). An overview of *Vibrio vulnificus* and *Vibrio parahaemolyticus*. Compr Rev Food Sci Food Safe.

[CR9] Hayat MZ, Kassu A, Mohammad A (2006). Isolation and molecular characterization of toxigenic *Vibrio parahaemolyticus* from the Kii Channel, Japan. Microbiol Res.

[CR10] Hervio-Heath D, Colwell RR, Derrien A (2002). Occurrence of pathogenic vibrios in coastal areas of France. J Appl Microbiol.

[CR11] Kaneko T, Colwell RR (1978). The annual cycle of *Vibrio parahaemolyticus* in Chesapeake Bay. Microb Ecol.

[CR12] Kelly MT, Stroh EMD (1988). Temporal relationship of *Vibrio parahaemolyticus* in patients and the environment. J Clin Microbiol.

[CR13] Martinez-Urtaza J, Lozano-Leon A, Varela-Pet J (2008). Environmental determinants of the occurrence and distribution of *Vibrio parahaemolyticus* in the Rias of Galicia, Spain. Appl Environ Microbiol.

[CR14] Nair GB, Ramamurthy T, Bhattacharya SK (2007). Global dissemination of *Vibrio parahaemolyticus* serotype O3:K6 and its serovariants. Clin Microbiol Rev.

[CR15] Okuda J, Ishibashi M, Abbott SL (1997). Analysis of the thermostable direct hemolysin (TDH) gene and the *tdh-*related hemolysin (*trh*) genes in urease-positive strains of *Vibrio parahaemolyticus* isolated on the West Coast of the United States. J Clin Microbiol.

[CR16] Pal D, Das N (2010). Isolation, identification and molecular characterization of *Vibrio parahaemolyticus* from fish samples in Kolkata. Eur Rev Med Pharmacol Sci.

[CR17] Tada J, Ohashi T, Nishimura N (1992). Detection of the thermostable direct hemolysin gene (*tdh*) and the thermostable direct hemolysin-related hemolysin gene (*trh*) of *Vibrio parahaemolyticus* by polymerase chain reaction. Mol Cell Probes.

[CR18] Velazquez-Roman J, Leon-Sicairos N, Flores-Villasenor H (2012). Association of pandemic *Vibrio parahaemolyticus* O3:K6 present in the coastal environment of Northwest Mexico with cases of recurrent diarrhea between 2004 and 2010. Appl Environ Microbiol.

[CR19] Yeung PS, Boor KJ (2004). Epidemiology, pathogenesis, and prevention of foodborne *Vibrio parahaemolyticus* infections. Foodborne Path Dis.

